# Patients' experiences of the quality of long-term care among the elderly: comparing scores over time

**DOI:** 10.1186/1472-6963-12-26

**Published:** 2012-01-31

**Authors:** Marloes Zuidgeest, Diana MJ Delnoij, Katrien G Luijkx, Dolf de Boer, Gert P Westert

**Affiliations:** 1 TRANZO, Scientific Centre for care and welfare, Faculty of Social and Behavioral Sciences, Tilburg University, PO Box 90153, 5000 LE Tilburg, the Netherlands; 2 Centre for Consumer Experience in Healthcare, P.O. Box 1568, 3500 BN Utrecht, the Netherlands; 3 NIVEL, Netherlands Institute for Health Services Research, PO Box 1568, 3500 BN Utrecht, the Netherlands; 4 IQ Healthcare, Scientific Institute for Quality of Healthcare, Radboud University Nijmegen Medical Centre, PO Box 9101, 114, 6500 HB Nijmegen, the Netherlands

## Abstract

**Background:**

Every two years, long-term care organizations for the elderly are obliged to evaluate and publish the experiences of residents, representatives of psychogeriatric patients, and/or assisted-living clients with regard to quality of care. Our hypotheses are that publication of this quality information leads to improved performance, and that organizations with substandard performance will improve more than those whose performance is relatively good.

**Methods:**

The analyses included organizational units that measured experiences *twice *between 2007 (t_0_) and 2009 (t_1_). Experiences with quality of care were measured with Consumer Quality Index (CQI) questionnaires. Besides descriptive analyses (i.e. mean, 5^th ^and 95^th ^percentile, and 90% central range) of the 19 CQI indicators and change scores of these indicators were calculated. Differences across five performance groups (ranging from 'worst' to 'best') were tested using an ANOVA test and effect sizes were measured with omega squared (ω^2^).

**Results:**

At t_0 _experiences of residents, representatives, and assisted-living clients were positive on all indicators. Nevertheless, most CQI indicators had improved scores (up to 0.37 change score) at t_1_. Only three indicators showed a minor decline (up to -0.08 change score). Change scores varied between indicators and questionnaires, e.g. they were more profound for the face-to-face interview questionnaire for residents in nursing homes than for the other two mail questionnaires (0.15 vs. 0.05 and 0.04, respectively), possibly due to more variation between nursing homes on the first measurement, perhaps indicating more potential for improvement. A negative relationship was found between prior performance and change, particularly with respect to the experiences of residents (ω^2 ^= 0.16) and assisted-living clients (ω^2 ^= 0.15). However, the relation between prior performance and improvement could also be demonstrated with respect to the experiences reported by representatives of psychogeriatric patients and by assisted-living clients. For representatives of psychogeriatric patients, the performance groups 1 and 2 ([much] below average) improved significantly more than the other three groups (ω^2 ^= 0.05).

**Conclusions:**

Both hypotheses were confirmed: almost all indicator scores improved over time and long-term care organizations for the elderly with substandard performance improved more than those with a performance which was already relatively good.

## Background

Various countries publish quality information about nursing homes and residential care facilities on the Internet in the form of report cards which serve multiple purposes [1-3]. First, quality information is available for choices by (future) clients or for families of these clients. Second, it can be used by nursing homes to account for their performance to healthcare regulators and government. Third, quality information informs health insurers about performance differences between nursing homes, which can be incorporated in their purchasing decisions. Finally, quality information can be used by nursing homes themselves to monitor and improve their quality of care [4]. In this article, the focus is on the latter function of public report cards, i.e. the role of transparency in quality improvement.

Countries differ in the quality information they provide on the report cards. Quality information can include *structure indicators *which refers to the conditions under which care is provided, *process indicators *which relates to the professional activities associated with providing care, and *outcome indicators *which denotes the effects of care [5]. Most countries provide the first two types of indicators and only some countries (Austria, Germany, the Netherlands, Sweden, USA) present the outcome measurements such as care-related safety, satisfaction, and experiences of residents or representatives [2].

In the Netherlands, the national indicator set for long-term care for the elderly is called the 'Quality Framework Responsible Care'[4]. It includes patient experience indicators which are measured with three separate questionnaires: a face-to-face interview protocol for residents in nursing homes, a mail questionnaire for representatives of psychogeriatric patients in residential care facilities, and a mail questionnaire for assisted-living clients that receive care from home care organizations. These questionnaires belong to the so-called Consumer Quality Index (CQI), which is the Dutch standard for measuring patient and client experiences in healthcare [6]. The national indicator set also includes clinical indicators like skin problems, depression, fall incidents, physical restraints, malnutrition, and medication errors. The present study focuses on indicators of patient experience.

Every organizational unit in long-term care (mostly a location of a nursing home, residential care facility or home care organization) is obliged to measure their performance on patient experience and clinical indicators. This obligation is defined by a national steering committee which includes representatives from client organizations, providers of long-term care for the elderly, healthcare regulators, the Dutch Ministry of Health, Welfare and Sport, and health insurers [7]. This national agreement is reinforced by the umbrella organization of nursing homes, homes for the elderly, and home care organizations, called ActiZ. Membership of ActiZ is terminated if members do not comply with the obligation to measure and publish client experiences. In addition, health insurers will cut the budget of healthcare providers who do not measure these indicators. These incentives spur care homes to measure and publish these scores. In 2007 (at the start of publishing the results), 62% of the organizational units published their outcomes [8] which increased to nearly all homes in 2010.

Providers can choose their own time at which they measure the experiences of residents, representatives and assisted-living clients, as long as they conduct the CQI surveys with an interval of about two years. Since 2007, several providers have measured and published their CQI survey results twice. In the present study we analyse CQI survey results of those providers in order to describe changes in their performance over time. Analyses of performance over time are made while taking into account all three CQI instruments for long-term care of the elderly. The results of those surveys are published on the level of indicators. Indicators correspond to the items and scales that were established during development of the questionnaires [9].

We hypothesize that publication of the CQI survey results of the first measurement (t_0_) will trigger activities to improve quality, which will lead to improved performance on the second measurement (t_1_). For example, in the USA care homes reorganized quality improvement programs and started new quality-assurance programs [10-12]. In addition, homes with poor quality scores were more likely to act on these performance scores than homes with better scores [10], a phenomenon that has also been observed in hospitals [13] and for health plans [14]. Thus, we hypothesize that organizations with substandard performance on the first measurement will show more improvement than organizations whose performance was already relatively good.

Our first research question is '*Have scores with respect to experiences of residents in nursing homes, representatives of psychogeriatric patients in residential care facilities, and assisted-living clients receiving care from home care providers improved between the first (t_o_) and the second (t_1_) measurement?' *Furthermore, investigation of scores takes place with respect to nursing homes, residential care facilities, and home care organizations that performed '(much) below average' on the first measurement compared with organizations that performed on 'average' and '(much) above average'. Therefore, the second research question is: *'Have nursing homes, residential care facilities, and home care providers that performed '(much) below average' improved more between t_0 _and t_1 _than those that performed 'average' and '(much) above average'?*

## Methods

### Sample

In the Netherlands, long-term care is generally provided at home or in nursing homes or residential homes (either in somatic or psychogeriatric wards or care units). These providers publish their CQI indicator scores in their Annual Reports (http://www.jaarverslagenzorg.nl). The dataset of indicator scores is publicly available. Between 2007 and 2009 a total of 499 organizational units (mostly a location of a nursing home, residential care facility or home care organization) had performed CQI surveys twice. Of these organizational units, 370 published CQI findings (at t_0 _and t_1_) of the face-to-face interviews with residents, 190 published findings of the mail questionnaire for representatives of psychogeriatric patients, and 122 published findings of the mail questionnaire for clients receiving care at home (assisted-living) questionnaires. One organizational unit can perform multiple questionnaires.

### Quality Framework Responsible Care

Client and clinical indicators belong to the Quality Framework 'Responsible Care' which encompasses seven quality domains. Four domains relate to quality of life: physical well-being and health, domestic and living conditions, participation and social handiness, mental well-being. Other domains are quality of caregivers, quality of care organization, and indicators with respect to more technical aspects of care (Table 1) [15].

**Table 1:** Seven themes and patient experience indicator scores (mean, 5^th ^and 95^th ^percentile, and the 90% central range) within the Quality Framework Responsible Care of the first measurement (t_0_)

	Questionnaire											
	**Residents**				**Represen-tatives**				**Assisted-living clients**			

**Indicator**	**Mean**	**P05**	**P95**	**90% range**	**Mean**	**P05**	**P95**	**90% range**	**Mean**	**P05**	**P95**	**90% range**

**1 Physical well-being and health**												

1_1 Body care	3.38	3.08	3.60	0.52	3.15	2.93	3.31	0.38	3.46	3.31	3.59	0.28

1_2 Meals	2.94	2.44	3.36	0.92	3.45	3.26	3.59	0.33	-	-	-	-

**2 Domestic and living conditions**												

2_1 Cleaning	3.28	2.75	3.63	0.88	3.14	2.70	3.49	0.79	-	-	-	-

2_2 Atmosphere	3.36	3.00	3.61	0.61	3.07	2.77	3.27	0.50	-	-	-	-

2_3 Housing and privacy	3.69	3.10	3.94	0.84	3.47	2.67	3.92	1.25	-	-	-	-

2_4 Experience of safety	3.72	3.55	3.83	0.28	2.80	2.5	3.05	0.55	3.43	3.26	3.55	0.29

**3 Participation and social handiness**												

3_1 Daily activities/participation	3.41	3.11	3.64	0.53	2.92	2.59	3.16	0.57	2.84	2.62	3.00	0.38

3_2 Autonomy	3.34	2.64	3.72	1.08	-	-	-	-	3.42	3.35	3.48	0.13

**4 Mental well-being**												

4_1 Mental well-being	3.18	2.96	3.37	0.41	3.22	3.01	3.39	0.38	3.37	3.30	3.45	0.15

**5 Quality of caregivers**												

5_1 Professionalism + safety care giving	3.43	3.14	3.67	0.53	3.28	3.09	3.4	0.31	3.51	3.35	3.63	0.28

5_2 Respectful treatment	3.37	3.06	3.62	0.56	3.46	3.29	3.58	0.29	3.58	3.42	3.67	0.25

5_3 Reliability caregivers	-	-	-	-	3.15	2.88	3.40	0.52	3.67	3.56	3.74	0.18

**6 Quality of care organization**												

6_1 Care plan + evaluation	3.16	2.60	3.66	1.06	3.41	3.19	3.63	0.44	3.65	3.56	3.71	0.15

6_2 Shared decision making	2.61	2.09	3.14	1.05	2.81	2.59	3.00	0.41	2.94	2.76	3.13	0.37

6_3 Information	2.74	2.23	3.17	0.94	3.25	2.88	3.56	0.68	3.16	2.99	3.32	0.33

6_4 Telephone accessibility	-	-	-	-	3.35	3.16	3.48	0.32	3.23	2.94	3.46	0.52

6_5 Coherence in care	-	-	-	-	-	-	-	-	3.08	2.80	3.34	0.54

6_6 Availability staff	2.92	2.51	3.26	0.75	2.97	2.66	3.17	0.51	3.25	2.97	3.41	0.44

**7 Technical aspects**												

7_12 Physical restraints	-	-	-	-	3.48	3.16	3.65	0.49	-	-	-	-

**Average score per questionnaire**	3.23	2.82	3.55	0.73	3.19	2.90	3.41	0.51	3.33	3.16	3.46	0.30

### CQI questionnaires and client indicators

Every two years, the actual experiences of residents are measured in a face-to-face interview, whereas experiences of family members and assisted-living clients are measured with mail questionnaires. Typically, response categories in the interview protocol and mail questionnaires refer to the frequency with which quality criteria were met: '1' never, '2' sometimes, '3' usually, and '4' always. The questionnaires belong to the CQI Long-term Care [9]. Collecting CQI data (e.g. conducting interviews, sending postal questionnaires and sending reminders), analysing and reporting the results is not done by the organizational units themselves, but can only be performed by certified survey vendors. Since this process takes considerable time and effort, data collection is possible during a two-year period. Data of all organizations were stored in a national databank.

### CQI indicators

**Figure 1:**
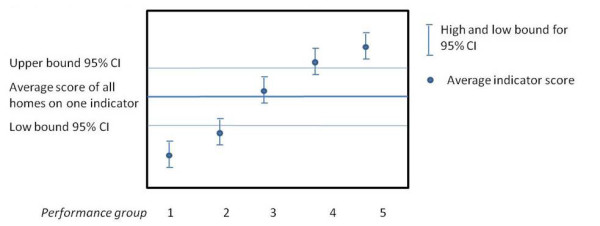
**Based on the indicator score and the 95% confidence interval, determination of the performance groups 1 ('worst') to 5 ('best')**.

The development of the CQI surveys and indicators is described elsewhere [16-18], including a report specifically focused on the CQI Long-term Care [9]. In brief, CQI indicators were determined by factor and reliability analysis [9,19]. The scores of the indicators were calculated for each respondent provided that half or more of the items were available. All indicator scores ranged from 1 to 4, where a score of '4' represents the 'best' rating. In total, 19 indicators (items and scales) were distinguished with the three questionnaires (Table 1). The analytical strategy used to compare CQI scores between healthcare providers is described elsewhere [20-22]. In brief, multi-level linear regression analyses (respondents were nested within homes) were performed to yield an empirical Bayes (EB) estimate per indicator and per organizational unit. The EB estimates an organization's mean indicator score, based on the scores within that particular organization and the distribution of the scores of all the other organizations. This method is widely recommended for analysis of institutional performance [22-24] and results in more stable estimates. In order to compare the EB estimates of organizations' indicator scores, so-called comparison intervals (calculated as ± 1.39 * SE) are used instead of 95% confidence intervals. These comparison intervals ensure that non-overlapping intervals of EB estimates represent a significant difference in indicator scores (p < 0.05) [25]. Apart from that, indicator scores were corrected for case-mix because client populations may differ on characteristics beyond the control of care providers [22,26]. For the interview protocol, the case-mix variables were age, education, perceived health, and length of stay. The indicator scores of the mail questionnaire to representatives were corrected for the kind of representative (e.g. spouse, son or daughter), residents' age and education, and length of stay. For the assisted-living clients questionnaire indicator scores were corrected for age, education, length of care, help with completing the questionnaire, and kind of care (cleaning house, personal care or assistant) [26]. The corrected indicator scores were divided into five performance groups after examining the data using: 1) the average score of all organizations on one indicator, 2) the average score of the higher bound of the comparison intervals, and 3) the average score of the lower bound of the comparison intervals. Figure 1 shows the classification of the performance groups representing '1' performance much below average (worst) to '5' representing performance much above average (best). We performed secondary analyses based on the corrected scores on two measurement points.

### Analysis

Per questionnaire, descriptive analyses on indicators scores (mean scores, the observed 5^th ^and 95^th ^percentile, and the 90% central range) were performed on the data of the first measurement (t_0_). The 90% central range is defined by the 5th and 95th percentiles to provide a stable measure of observed dispersion. This is an indication of potential for improvement, because greater dispersion means that lower performing organizations can make more improvement, as there will be a larger difference with higher performing organizations. A change score per indicator was calculated by subtracting the indicator scores between those two measurement points (t_0 _- t_1_). Per CQI questionnaire and per indicator we analysed these change scores. In addition, we examined the overall change scores per questionnaire for different so-called performance groups. Performance groups refer to the performance scored at t_0 _and ranged from 1 (much below average) to 5 (much above average) (Figure 1). Differences in change scores across performance groups were tested using an ANOVA test. If the assumption of equal variances was violated, the Welch test of robust test of equality of means was used. Tukey post-hoc tests were used to compare the mean difference score of every performance group to the means of every other performance group, and identifies where the differences between two means is greater than the standard error would be expected. Effect sizes were measured with omega squared (ω^2^) [25]. Omega squared estimates the proportion of variance explained for the population and is calculated as ((SS_Between_-(df_Between _*MS_Within_))/(SS_Total_+MS_Within_). A small, medium and large effect are defined as ω^2 ^= 0.01, 0.06 and 0.14, respectively [27]. A p-value < 0.05 was considered statistically significant. Analyses were performed using SPSS version 17.0.

## Results

Table 1 presents per indicator and per questionnaire the mean scores, the 5^th ^and 95^th ^percentile, and the 90% central range. At t_0 _the experiences of respondents on all indicators were positive for the interview questionnaire, as were the experiences of representatives and assisted-living clients on the mail questionnaire: 3.23 (2.82-3.55), 3.19 (2.90-3.41), and 3.33 (3.16-3.46), respectively. For the interview questionnaire the 90% range was 0.73, and for the mail questionnaires (representatives and assisted-living clients) it was 0.51 and 0.30, respectively.

The largest difference between the 5^th ^and 95^th ^percentile of the interview questionnaire was for the indicators 3_2 'Autonomy' (1.08), 6_1 'Care plan and evaluation' (1.06), and 6_2 'Shared decision making' (1.05). Housing and privacy (indicator 2_3) is an indicator of the representatives' questionnaire that showed considerable variation between organizational units (1.25). No indicators of the third questionnaire diverged as much as the other questionnaires. Indicator 6_5 'Coherence in care' showed the most variation (0.54).

**Figure 2:**
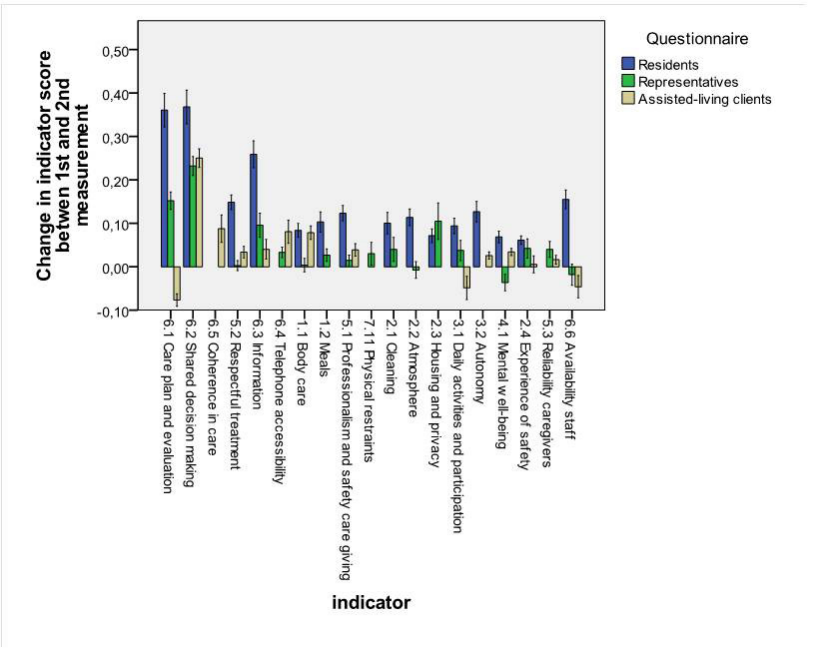
**Change scores of all indicators and the 95% confidence interval**.

Figure 2 shows the change scores per indicator for all three questionnaires. We expected change scores to be limited by the 90% observed range on the first measurement; this range varied between indicators and questionnaires (Table 1). Theoretically, an indicator score can change 3 points (from 1 to 4, and vice versa). The change scores for the interview questionnaires were all positive (0.06-0.37). For the interviews with residents and the mail questionnaire for representatives, the three indicators that improved the most were the indicators 6_2 'Shared decision making' (0.37; 0.23), 6_1 'Care plan and evaluation' (0.36; 0.15), and 6_3 'Information' (0.26; 0.10). Indicator 2_3 'Housing and privacy' also changed by 0.10 for the representatives questionnaire. Scores of the questionnaire for assisted-living clients improved the most with respect to indicators 6_2 'Shared decision making' (0.25), 6_5 'Coherence in care' (0.09), and 6_4 'Telephone accessibility'(0.08). The surveys of the representatives showed a decline in indicator scores 4_1 'Mental well-being' (-0.04), 6_6 'Availability staff' (-0.02), and 2_2 'Atmosphere' (-0.01). In the assisted-living clients questionnaire, three indicators showed a decline: indicators 6_1 'Care plan and evaluation' (-0.08), 3_1 'Daily activities' (-0.05), and 6_6 'Availability staff' (-0.05). The change score of the assisted-living clients (range -0.08 to 0.25) did not diverge to the same extent as the change scores of the other questionnaires.

**Figure 3:**
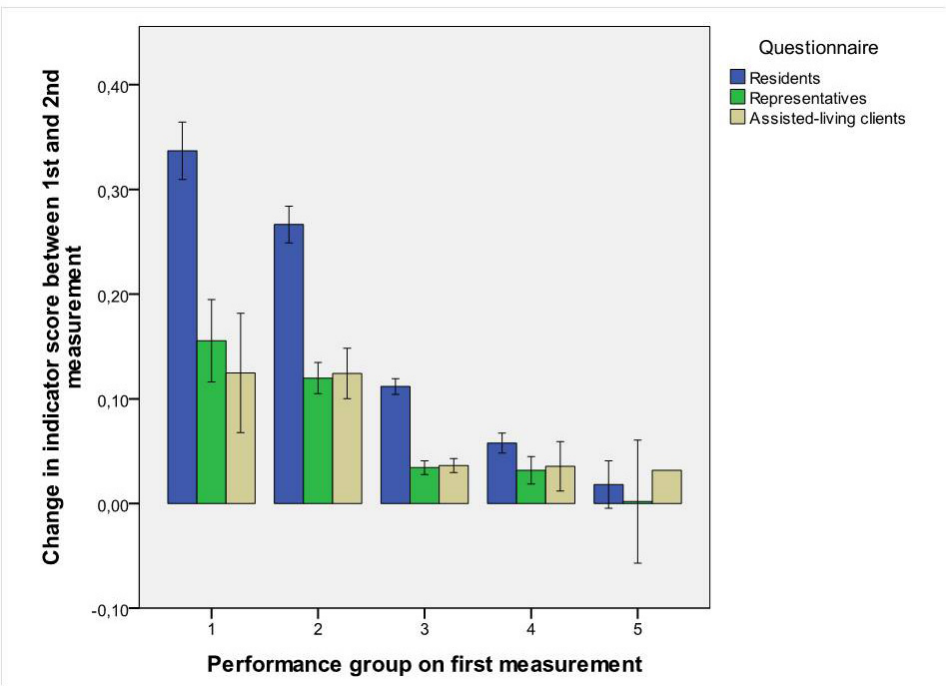
**Mean difference in average score on all indicators and the 95% confidence interval**.

To illustrate the differences in scores between questionnaires, Figure 3 shows the mean change scores over all indicators between the two measurement points (t_0 _and t_1_). As expected, there was a negative relationship between prior performance and change (Figure 3). At t_1 _the organizational units of performance groups 1, 2 and 3 showed an average improvement of 0.34 (SD = 0.29), 0.27 (0.23), and 0.11 (0.20), respectively. At t_1 _the organizational units of performance groups 4 and 5 showed an average improvement of 0.06 (0.12) and 0.02 (0.12), respectively.

Change scores of the indicators based on the interview questionnaire changed to a greater extent than those of the other two questionnaires: all scores between the performance groups showed a significant difference (*p *< 0.001) and the effect was large (ω^2 ^= 0.16). Regarding the questionnaire for representatives there was a significant difference between the performance groups (*p *< 0.001), which was a medium effect (ω^2 ^= 0.05). Performance groups 1 and 2 differed from all the other performance groups. Performance groups 3, 4 and 5 did not differ from each other (*p *> 0.05). For the questionnaire for assisted-living clients there was a significant difference between all four performance groups (*p *< 0.001), which was a large effect (ω^2 ^= 0.15). Performance group 5 was excluded from the analysis, because there was only one indicator in group 5 at t_0_.

**Figure 4:**
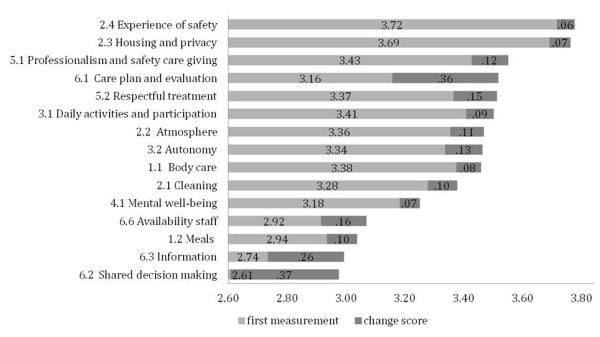
**Per indicator, the average score of the first measurement (t_0_) and the change score (t_1 - _t_0_) for the face-to-face interview questionnaire for residents**.

Combining change scores with the scores at the first measurement (t_0_) provides insight into the improvement potential per indicator. To illustrate this, Figure 4 shows the scores at t_0 _together with the change scores (t_1 _- t_0_) for the indicators based on the interview questionnaire. The same information for the indicators can be constructed for the other two questionnaires (presented in Additional file 1), but the main findings of both figures are described. The three highest indicator scores at the second measurement (t_1_) were for indicators 2_4 'Experience of safety' (3.78), 2_3 'Housing and privacy' (3.76) and 5_1 'Professionalism and safety care giving' (3.55). The three highest scores of the questionnaire for representatives were for the indicators 2_3 'Housing and privacy' (3.58), 6_1 'Care plan and evaluating' (3.56), and 7_12 'Physical restrains' (3.51). Finally, the three highest scores for the assisted-living clients were for the indicators 5_3 'Reliability caregivers' (3.69), 5_2 'Respectful treatment' (3.61), and 6_1 'Care plan and evaluation' (3.57).

Although indicator 6_2 'Shared decision making' changed the most between the t_0 _and t_1_, the score at t_1 _remained the lowest (2.98) of the interview questionnaire. Indicators 6_3 'Information', 1_2 'Meals', and 6_6 'Availability staff' are ones that can make the most improvement in a future measurement (2.99, 3.04, 3.07, respectively), also because their 5^th ^and 95^th ^percentile range shows differences between the care homes (> 0.75 range score).

Indicator 2_4 ('Experience of safety') based on questionnaires for representatives/family members barely changed between t_0 _and t_1_. The score on indicator 6_2 'Shared decision' increased considerably, but is still one of the lowest indicator scores. Indicators that scored high at t_0 _and remained high at t_1 _were indicators 2_3 'Housing and privacy', 6_1 'Care plan and evaluation', 7_11 'Physical restrains', and 1_2 'Meals'.

Overall, the indicator scores of the questionnaire for assisted-living clients showed only minor changes (0.01-0.09). An exception to this was the indicator 6_2 ('Shared decision making'), which changed the most (0.25) but still had one of the lowest scores (2.94). Indicators scoring low at t_0 _and remaining low were the indicators 3_1 'Daily activities and participation' (2.84) and 6_5 'Coherence in care' (3.08).

## Discussion

This study investigated patients' experiences of the quality of long-term care among the elderly. More specifically, we looked at scores over time to evaluate whether indicator scores of nursing homes, residential care facilities and homecare providers, had improved and whether healthcare organizations that performed (much) below average improved more than organizations that performed on average and (much) above average.

Our first hypothesis was that publishing the CQI survey results of the first measurement (t_0_) would trigger quality improvement activities which would lead to improved performance at the second measurement (t_1_). Our results confirm that most indicator scores improved at t_1_. Performance improved the most with respect to topics on the quality domain 'Quality of care organization'. Indicators belonging to this quality domain are *care plan, shared decision making*, and *coherence in care*. None of the indicator scores based on interviews with residents showed a decrease. The indicators based on mail questionnaires for representatives/assisted-living clients showed a decline of scores for three indicators. In addition, indicators that showed more dispersion of the 90% observed range at the first measurement also showed more improvement at the second measurement. This suggests that indicators with scores having a large range can more easily show improvement.

A preliminary Dutch report failed to show an improvement in scores [28]; this might be because the authors analysed a much smaller sample of organizational units and used data on the client level whereas our study was based on overall average scores per indicator per organizational unit. Moreover, data in that study missed the variance per indicator scores for comparison over time; nevertheless, the calculated scores of both measurement points are based on the original corrected scores with the confidence intervals [28]. On the other hand, our findings are consistent with other international reports, as well as with theories on quality management and quality improvement [10,13,14]. Studies in the USA have shown that homes reorganized quality improvement programs and started new quality-assurance programs in response to public performance scores [10,12,29]. Although there are no Dutch reports on specific actions taken by nursing homes, residential care facilities or homecare organizations to improve their scores, one qualitative study indicated that organizations start various quality improvement activities in response to the CQI results [30].

When examining specific changes in indicator scores, we see that the indicator '*Care plan and evaluation*' improved. One explanation for this is the modification made in the method of data collection of the interview protocol. Explaining the term '*care plan' *was not allowed at t_0 _whereas at t_1 _the interviewers explained that '*care plan*' could be the '*green folder*', '*red folder*', or '*care-living plan*', or whatever the care plan was called in that specific organizational unit. Another explanation for the improvement of indicator scores is involvement of the health insurer. If insurers indicate that a certain quality aspect is important and reward higher scores, organizations will probably improve more on those quality aspects. For the *care plan*, health insurers also have their own teams that perform administrative controls and trigger improvement in this area [30]. The change scores of some indicators of the mail questionnaires for representatives and assisted-living clients are close to zero or even slightly negative. This could imply that changes in these areas are difficult to accomplish within a two-year period.

The second hypothesis stated that nursing homes, residential care facilities, and homecare providers with a substandard performance at t_0 _will show more improvement than those organizations whose performance was already relatively good. The overall change scores for the five performance groups showed that healthcare organizations in group 1 improved more than organizations in group 5. This relationship was stronger for the indicators based on the interview questionnaire with residents than for the indicators based on the mail questionnaires. For the mail questionnaire for representatives, performance groups 3, 4 and 5 did not differ significantly from each other regarding changes over time. These results are similar to those of Mukabel et al. who also found that care homes with poor quality scores were more likely to act on performance scores compared with those with better scores [10]. This might be related to the sense of urgency that managers/professionals in poor performing organizations may experience. For instance, Baier et al. found that homes with ambitious targets improve more than homes with less-ambitious targets [30]. More research is needed to elucidate the responses of nursing home staff/board members to the CQI results in terms of strategic orientation [31]. In the future, pay-for-performance may be a mechanism through which quality improvement can be achieved. In 2010, some healthcare purchasers have started to use CQI scores along with a number of other criteria to determine prices in long-term care. However, for individual care providers the financial consequences of performing low on CQI indicators, or of not publishing their results on the Internet were extremely limited.

### Limitations

The present study has several limitations.

First, the response categories in the interview protocol and questionnaires range from '1' never' to '4' always. This means that there is a ceiling effect when the frequency of the quality criteria is consistently good (score is 4). Some indicators reached this ceiling at t_0_, implying that there was little opportunity for improvement. For example, at t_1 _the indicator 'Experience of safety' of the interview questionnaire had a score of 3.78 at t_1 _and indicator 'Housing and privacy' had a score of 3.76, indicating that the experiences of residents were very positive and that poorer performing organizational units had more room for improvement. This ceiling effect was more profound for the interview questionnaire than for the mail questionnaires. This may be due to a mode effect, because (telephone) interviewees are reported to respond more positively than mail respondents [32].

Finally, the indicator score represents a case-mix corrected score per healthcare organization based on data on the level of the individual client. For the present study, we only had access to the average indicator scores per organizational unit. Thus, we could analyse improvement over time on the level of the organizational unit, looking at different groups of organizations and aggregated indicator scores. However, because data at the client level were not provided by the national databank, it was not possible examine the variance of the indicator scores on the client level. Future studies should explore changes over time using data on the client level nested in organizational units. To analyse changes over time per organizational unit, multilevel analysis is necessary with: i) patients, ii) nested in organizations, and iii) available per year. Use of this data processing method will allow to determine whether single organizational units have significantly improved over time.

Finally, we analysed the differences in performance between t_0 _and t_1_. Although we assume that organizational units have engaged in quality improvement strategies between these two points, we had no details on such quality improvement activities. Also, we have no data on the differences in the characteristics of respondents the between the two measurement point. It seems unlikely that the changes in performance between t0 and t1 were influenced by differences in case-mix as only data was used that were corrected for casemix.

## Conclusions

Comparison of long-term care indicator scores over time revealed that indicator scores did improve. Nursing homes, residential care facilities, and homecare providers with substandard performance on the first measurement showed more improvement than those organizations whose performance was already relatively good.

## Competing interests

The authors declare that they have no competing interests.

## Authors' contributions

MZ analysed and interpreted the data, and wrote a draft manuscript. DD, DdB, KL, and GW made critical revisions to the manuscript. All authors have read and approved the final manuscript.

## Pre-publication history

The pre-publication history for this paper can be accessed here:

http://www.biomedcentral.com/1472-6963/12/26/prepub

## Supplementary Material

Additional file 1 **Figures of the mail questionnaire for representatives and assistend-living clients**. Per indicator, the average score of the first measurement (t_0_) and the change score (t_1 _- t_0_) of the two different mail questionnaires are displayed. Click here for file
