# Supplementary material for: Patients' experiences of the quality of long-term care among the elderly: comparing scores over time
Source: BMC Health Serv Res. 2012 Jan 31;12:26. doi: 10.1186/1472-6963-12-26 (PMC3305532; doi:10.1186/1472-6963-12-26)

**Appendix**

Figure 1 Per indicator, the average score of the first measurement (t_0_) and the change score (t_1_ - t_0_) for the mail questionnaire for representatives.
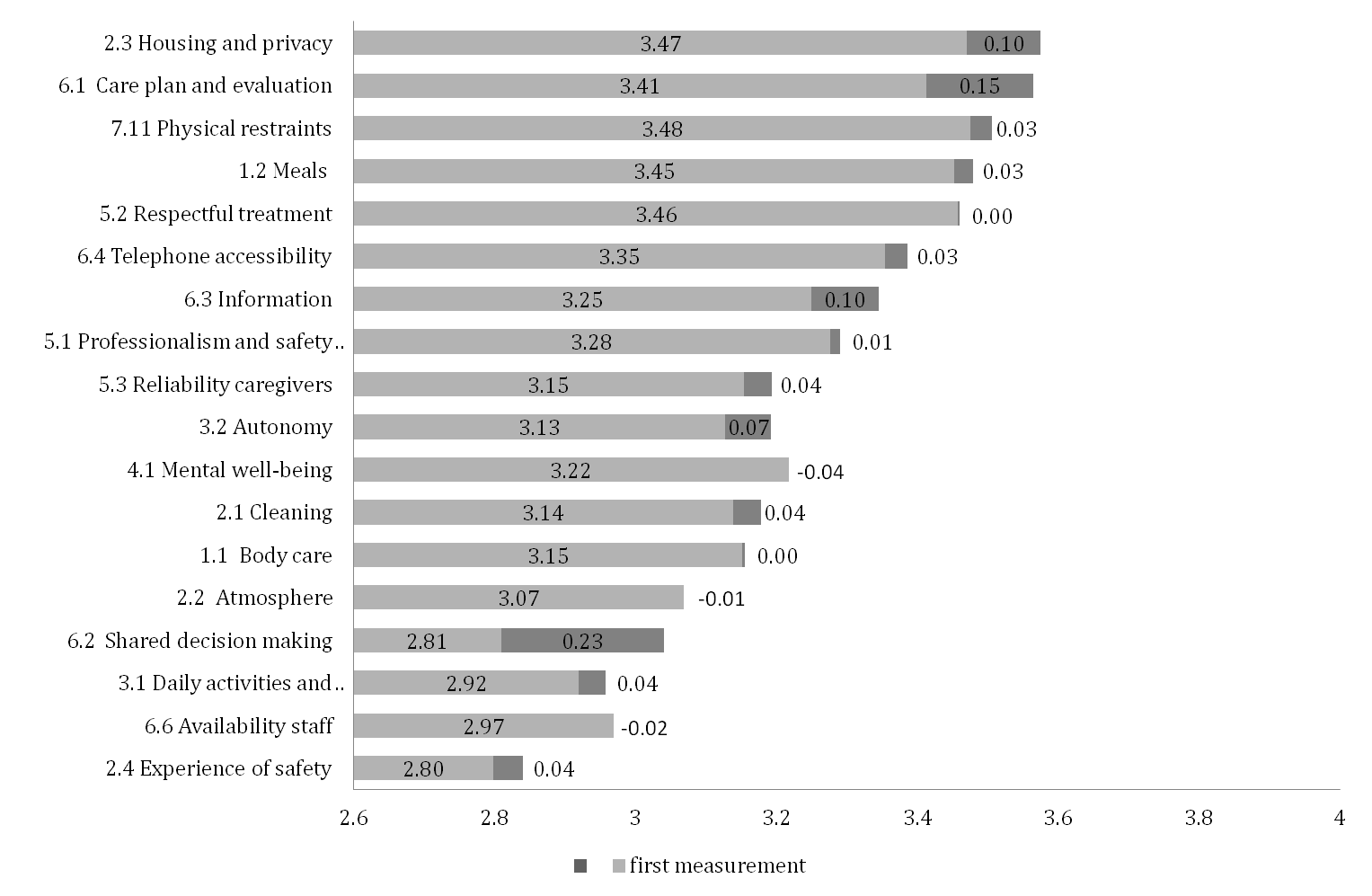


Figure 2 Per indicator, the average score of the first measurement (t_0_) and the change score (t_1_ - t_0_) for the mail questionnaire to assisted-living clients.
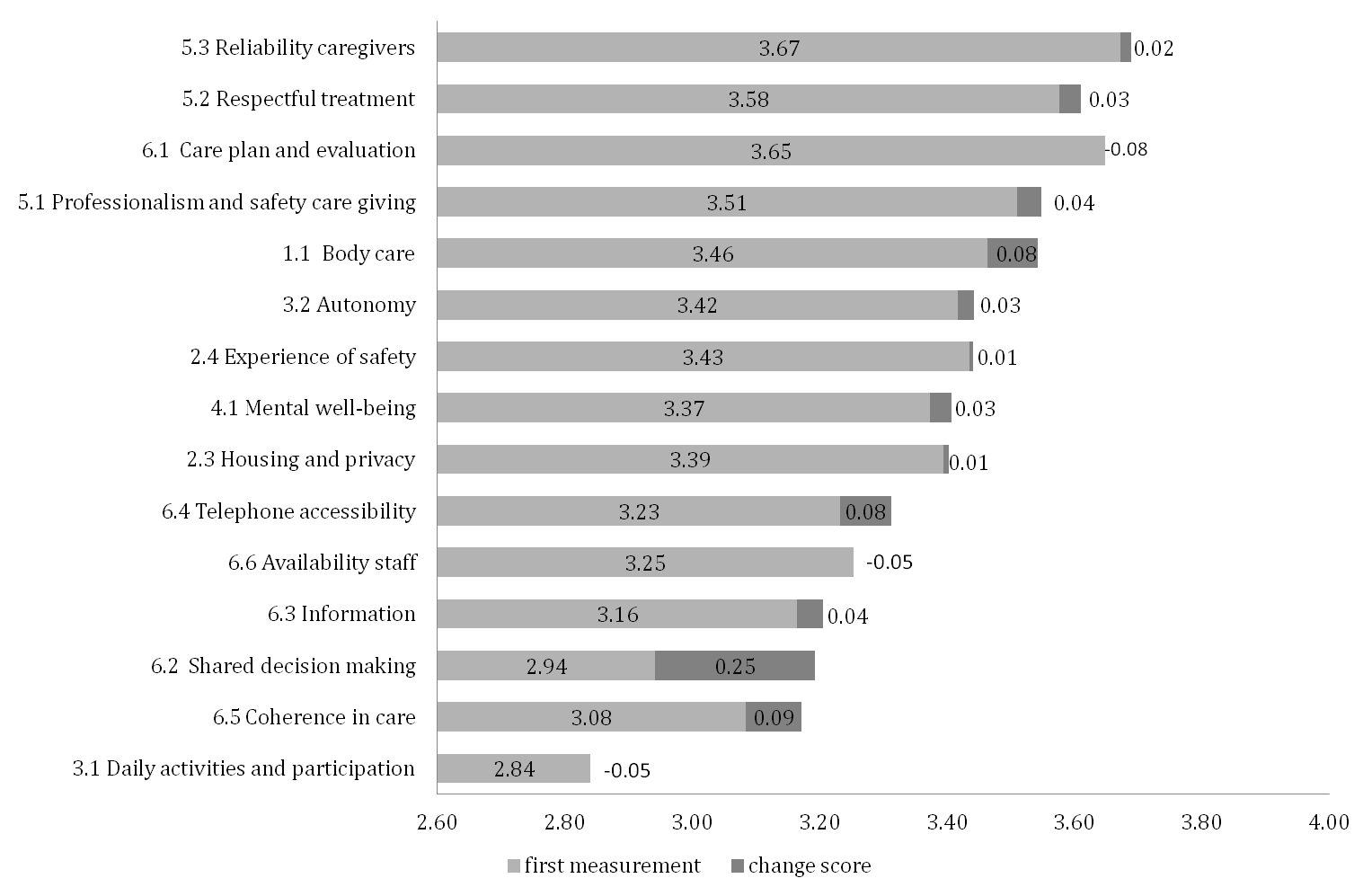

Supplement: Additional file 1 — Figures of the mail questionnaire for representatives and assistend-living clients. Per indicator, the average score of the first measurement (t0) and the change score (t1 - t0) of the two different mail questionnaires are displayed. [file 1472-6963-12-26-S1.DOC]
